# Advancing rheumatoid arthritis care: the role of genetics and personalized medicine

**DOI:** 10.1097/MS9.0000000000003969

**Published:** 2025-09-23

**Authors:** Rano Mal, Vijay Singh, Muhammad Hassan Javaid, Muddassir Khalid, Fred Segawa

**Affiliations:** a Department of Medicine, I Liaquat University of Medical & Health Sciences, Jamshoro, Pakistan; b Department of Medicine, Shifa College of Medicine, Islamabad, Pakistan; c Department of Medicine, Nishtar Medical University, Multan, Pakistan; d Department of Medicine, Makerere University School of Health Sciences, Kampala, Central Region, Uganda

*Dear Editor,*

A common autoimmune disease, rheumatoid arthritis (RA), is characterized by systemic effects and persistent inflammatory arthritis. The complicated etiology of RA includes environmental variables, especially smoking, which increases the risk of the disease, as well as genetic factors, such as HLA-DR4 and HLA-DR1. Dietary changes, exercise, and stress reduction are nonpharmacological strategies that are becoming more and more regarded for their complementary roles in managing RA[1]. Two of the Janus kinase (JAK) inhibitors that are currently on the market for the treatment of RA patients are baricitinib and facitinib. According to randomized controlled trials, these JAK inhibitors work just as well as biological disease-modifying antirheumatic drugs (DMARDs). Although the safety profiles of these JAK inhibitors have been proven in randomized controlled trials and their long-term extension studies, empirical evidence is still needed to close the gap between rheumatology clinics and randomized controlled trials[2].

Most (>80%) of the known heritability in each community (4.7% for Europeans and 3.8% for Asians) may be explained by the trans-ethnic genetic risk model, which is based on Risk Adjustment Factor from one population but OR from the other. These findings provide credence to our theory that Asians and Europeans generally share a genetic predisposition to RA[3]. Health-Related Quality of Life (HR-QoL) is significantly impacted by RA. This backs up current National Institute for Health and Care Excellence guidelines that state RA patients should have regular evaluations to see how their disease affects their HRQoL and receive the proper care[4]. Advances in the treatment of RA are the result of two simultaneous breakthroughs in recent decades. First, advances in biotechnology and a better understanding of the pathobiology of the disease have led to an expanded arsenal of targeted treatments. Second, titrating treatments, also known as the treat-to-target strategy, has been ushered in by the recognition that early and efficient control of inflammation enhances results[5].

Pharmacological methods to discovering a cure for RA have advanced significantly as a result of ongoing improvements in drug design tactics and procedures. The symptoms have been lessened, the progression has been slowed, and consequences have been avoided thanks to the new treatment alternatives. According to ACR and EULAR (European Alliance of Associations for Rheumatology) guidelines, the two main approaches to managing RA are disease-modifying management (DMARDs) and symptomatic treatment (NSAIDs and GCs)[6].

The etiology of RA is complex, similar to that of many other autoimmune disorders. Familial clustering and monozygotic twin studies show genetic vulnerability, with genetic variables accounting for 50% of the risk of RA. Human leukocyte antigens -DR45 and -DRB1, as well as a number of alleles known as the common epitope, are genetically linked to RA. Additional genetic markers, such as the STAT4 gene and CD40 region, have been found through genome-wide association studies to raise the risk of RA and other autoimmune illnesses. The main environmental cause of RA, particularly in people with a genetic susceptibility, is smoking. No specific pathogen has been identified as the cause of RA, albeit infections may reveal an inflammatory reaction[7].

TITAN Guidelines: This manuscript complies with TITAN Guidelines, 2025, declaring no use of AI[8].

## Data Availability

The data used during the current study are available from the corresponding author on reasonable request.
